# Supplementary material for: Pilot study of large-scale production of mutant pigs by ENU mutagenesis
Source: eLife. 2017 Jun 22;6:e26248. doi: 10.7554/eLife.26248 (PMC5505698; doi:10.7554/eLife.26248)
Supplement: Supplementary file 1. — DOI: http://dx.doi.org/10.7554/eLife.26248.016 [file elife-26248-supp1.docx]

**Supplementary file 1.** Summary of the 2b-RAD sequencing analysis

| **Samples** | **Raw reads** | **Processed reads ^a^** | **Ratios of**  **processed reads** | **RAD-tag** |
| --- | --- | --- | --- | --- |
| 26 | 7,884,253 | 7,508,035 | 95.2% | 379,554 |
| B08 | 8,229,307 | 7,684,923 | 93.4% | 441,317 |
| T113 | 7,962,281 | 6,912,344 | 86.8% | 462,545 |
| T114 | 6,238,969 | 5,653,180 | 90.6% | 422,344 |
| T115 | 9,012,397 | 8,184,358 | 90.8% | 470,561 |
| T116 | 9,262,722 | 7,907,662 | 85.4% | 486,805 |
| T117 | 9,522,242 | 8,658,077 | 90.9% | 468,860 |
| 6 | 8,435,825 | 8,059,891 | 95.5% | 399,670 |
| 53 | 8,908,285 | 8,119,131 | 91.1% | 446,597 |
| T001 | 8,580,073 | 8,224,607 | 95.9% | 389,640 |
| T002 | 7,653,448 | 7,305,756 | 95.5% | 387,434 |
| T003 | 6,517,954 | 6,209,811 | 95.3% | 385,387 |
| T004 | 7,462,540 | 7,126,924 | 95.5% | 389,315 |
| T005 | 7,023,051 | 6,644,289 | 94.6% | 402,145 |

^a^ Reads were filtered using the following thresholds: reads without restriction sites, reads containing long homo-polymers (more than 10 bp) or ambiguous bases (N) and reads with many low-quality bases (more than 10 positions with quality rated less than 20).
