# Supplementary material for: Pilot study of large-scale production of mutant pigs by ENU mutagenesis
Source: eLife. 2017 Jun 22;6:e26248. doi: 10.7554/eLife.26248 (PMC5505698; doi:10.7554/eLife.26248)
Supplement: Supplementary file 2. — DOI: http://dx.doi.org/10.7554/eLife.26248.017 [file elife-26248-supp2.docx]

**Supplementary file 2.** Summary of the sequenced ENU-induced mutations in two families

| **Family** | **Pig ID** | **ENU dose** | **G1** | **Sites^a^ (bp)** | **Mutations^b^** | **Frequency^c^** | **Average** |
| --- | --- | --- | --- | --- | --- | --- | --- |
| Family 1 | Boar: 26  Sow: B08 | 65 mg/kg | T113 | 4047197 | 9 | 2.22E-06 | 1.63E-06 |
|  |  |  | T114 | 3548561 | 8 | 2.23E-06 |  |
|  |  |  | T115 | 4315382 | 5 | 1.16E-06 |  |
|  |  |  | T116 | 4234604 | 5 | 1.18E-06 |  |
|  |  |  | T117 | 4474832 | 6 | 1.34E-06 |  |
| Family 2 | Boar: 6  Sow: B53 | 85 mg/kg | T001 | 4122798 | 13 | 3.15E-06 | 5.86E-06 |
|  |  |  | T002 | 4547486 | 38 | 8.36E-06 |  |
|  |  |  | T003 | 4363692 | 23 | 5.27E-06 |  |
|  |  |  | T004 | 4044881 | 31 | 7.66E-06 |  |
|  |  |  | T005 | 4317205 | 21 | 4.86E-06 |  |

# ^a^ To screen the ENU-induced mutations, ten trios (father-mother-child) were analyzed. Based on the 2b-RAD sequencing data, genomic sites covered by at least 10 reads in any member of the trio were recorded and could be used for subsequent analysis.

# ^b^ ENU-induced mutations were analyzed using DeNovoGear software.

# ^c^ Frequency = mutations/sites.
