# Supplementary material for: Pilot study of large-scale production of mutant pigs by ENU mutagenesis
Source: eLife. 2017 Jun 22;6:e26248. doi: 10.7554/eLife.26248 (PMC5505698; doi:10.7554/eLife.26248)
Supplement: Supplementary file 3. — DOI: http://dx.doi.org/10.7554/eLife.26248.018 [file elife-26248-supp3.docx]

| **Groups** | **Phenotype** | **Number** | **AA** | **AT** | **TT** | **A%** | **T%** |
| --- | --- | --- | --- | --- | --- | --- | --- |
| TBB007T095 line | Mutant | 63 | 0 | 63 | 0 | 50% | 50% |
|  | WT | 71 | 71 | 0 | 0 | 100% | 0 |
| Bama | WT | 38 | 38 | 0 | 0 | 100% | 0 |
| Large White | WT | 53 | 53 | 0 | 0 | 100% | 0 |
| Landrace | WT | 34 | 34 | 0 | 0 | 100% | 0 |
| Duroc | WT | 37 | 37 | 0 | 0 | 100% | 0 |

**Supplementary file 3.** The frequency and distribution of *SOX10* c. 325 A>T mutation among TBB007T095 Lines and other breeds
