# Supplementary material for: Pilot study of large-scale production of mutant pigs by ENU mutagenesis
Source: eLife. 2017 Jun 22;6:e26248. doi: 10.7554/eLife.26248 (PMC5505698; doi:10.7554/eLife.26248)
Supplement: Supplementary file 4. — DOI: http://dx.doi.org/10.7554/eLife.26248.019 [file elife-26248-supp4.docx]

**Supplementary file 4.** Genes located in the linkage region of chr4: 16-17 Mb

| **Genes ^a^** | **Chr** | **Position:start** | **Position:stop** | **Gene functional annotation** |
| --- | --- | --- | --- | --- |
| *LOC102157933* | 4 | 15986366 | 16001214 | uncharacterized LOC102157933 |
| *LOC102159002* | 4 | 16078179 | 16082538 | uncharacterized LOC102159002 |
| ***FER1L6*** | 4 | 16140979 | 16244794 | fer-1 like family member 6 |
| *FAM91A1* | 4 | 16359647 | 16401246 | family with sequence similarity 91 member A1 |
| *LOC102161657* | 4 | 16385813 | 16388877 | 40S ribosomal protein S3a-like |
| *LOC102159439* | 4 | 16407108 | 16410962 | uncharacterized LOC102159439 |
| ***ANXA13*** | 4 | 16448340 | 16485953 | annexin A13 |
| *LOC102159893* | 4 | 16488060 | 16507529 | uncharacterized LOC102159893 |
| ***KLHL38*** | 4 | 16543629 | 16545876 | Kelch-like family member 38 |
| ***FBXO32*** | 4 | 16639187 | 16669235 | F-box protein 32 |
| ***WDYHV1*** | 4 | 16724003 | 16742260 | WDYHV motif containing 1 |
| *LOC102160625* | 4 | 16748552 | 16755779 | uncharacterized LOC102160625 |
| ***ATAD2*** | 4 | 16755848 | 16825159 | ATPase family, AAA domain containing 2 |
| *LOC102160714* | 4 | 16837750 | 16839235 | uncharacterized LOC102160714 |
| ***ZHX1*** | 4 | 16844907 | 16870794 | zinc fingers and homeoboxes 1 |
| *LOC102160796* | 4 | 16851448 | 16854623 | uncharacterized LOC102160796 |
| *C4H8orf76* | 4 | 16881291 | 16905854 | chromosome 4 open reading frame, human C8orf76 |
| *FAM83A* | 4 | 16913828 | 16928901 | family with sequence similarity 83 member A |
| ***TRNAM-CAU*** | 4 | 16940605 | 16940677 | transfer RNA methionine (anticodon CAU) |
| ***TBC1D31*** | 4 | 16999345 | 17046947 | TBC1 domain family member 31 |

^a^ Genes with characterizations or functional annotations are shown in bold.
